# Decreased PTGES2 Farnesylation in Granulosa Cells Compromises PGE2‐Dependent Cumulus Expansion and Oocyte Maturation During Ovarian Aging

**DOI:** 10.1111/acel.70374

**Published:** 2026-01-11

**Authors:** Sainan Zhang, Jiahui Qi, Chuanming Liu, Huidan Zhang, Bichun Guo, Die Wu, Yicen Liu, Xin Zhen, Yang Zhang, Nannan Kang, Jidong Zhou, Guijun Yan, Chaojun Li, Lijun Ding, Haixiang Sun

**Affiliations:** ^1^ Center for Reproductive Medicine and Obstetrics and Gynecology, Nanjing Drum Tower Hospital Clinical College of Nanjing Medical University Nanjing China; ^2^ State Key Laboratory of Reproductive Medicine and Offspring Health Nanjing Medical University Nanjing China; ^3^ Center for Reproductive Medicine and Obstetrics and Gynecology, Nanjing Drum Tower Hospital, Affiliated Hospital of Medical School Nanjing University Nanjing China; ^4^ Center for Reproductive Medicine and Obstetrics and Gynecology, Joint Institute of Nanjing Drum Tower Hospital for Life and Health, College of Life Science Nanjing Normal University Nanjing China; ^5^ Center for Molecular Reproductive Medicine Nanjing University Nanjing China; ^6^ Jiangsu Human Reproductive Function Remodeling Engineering Research Center Nanjing China; ^7^ Clinical Center for Stem Cell Research, Nanjing Drum Tower Hospital Nanjing University Nanjing China; ^8^ State Key Laboratory of Analytic Chemistry for Life Science Nanjing University Nanjing China

**Keywords:** cumulus expansion, farnesylation, oocyte maturation, ovarian aging, PTGES2

## Abstract

With the increasing trend of delayed childbearing, the decline in oocyte quality associated with advanced maternal age has emerged as a pressing concern. However, the mechanism remains unclear, and effective strategies for improvement are currently lacking. Previously, we reported that the downregulation of the mevalonate pathway in aged granulosa cells (GCs) contributed to meiotic defects in oocytes, which may implicate farnesyl pyrophosphate‐mediated protein farnesylation. Nevertheless, the role of farnesylation in ovarian aging and its impact on oocytes requires further investigation. In this study, using cumulus‐oocyte complexes (COCs) from young and aged female mice, we observed impaired cumulus expansion and concurrent meiotic defects during aged oocyte maturation, accompanied by significantly reduced protein farnesylation in aged GCs. Furthermore, inhibiting farnesylation with FTI‐277 in young COCs recapitulated the aging phenotype, disrupting cumulus expansion and inducing meiotic defects similar to those in aged COCs. Conversely, restoring farnesylation via farnesol supplementation effectively ameliorated these deficits in both aged COCs (in vitro) and aged mice (in vivo). Proteomic analysis and experimental validation identified prostaglandin E2 synthase 2 (PTGES2) as a farnesylated protein. Mechanistically, age‐related decline in PTGES2 farnesylation in GCs reduces its endoplasmic reticulum localization and impairs prostaglandin E2 (PGE2) production, thereby compromising PGE2‐dependent cumulus expansion and oocyte maturation. Collectively, our findings highlight the detrimental effects of decreased farnesylation in aged GCs on oocyte quality and propose a potential therapeutic strategy for improving the developmental competence of aged oocytes.

## Graphical abstract

A schematic showing decreased PTGES2 farnesylation in granulosa cells compromises PGE2‐dependent cumulus expansion and oocyte maturation during ovarian aging. In young ovaries, PTGES2 farnesylation of GCs regulates the process of cumulus expansion and oocyte maturation by facilitating PGE2 production. With aging, the downregulation of PTGES2 farnesylation in GCs disrupts PTGES2 localization to the ER, thereby impairing cumulus expansion and oocyte maturation through reduced PGE2 production.

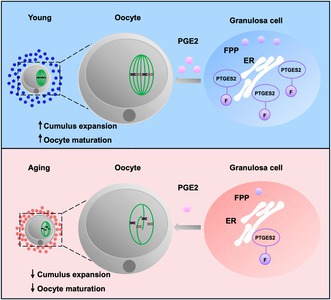

## 1 Introduction

Over recent decades, the trend towards postponement of childbearing has significantly emerged and led to increased fertility needs among women of advanced reproductive age (Ding et al. 2025; Waldenström 2016). Advancing age correlates with reduced fecundity and increased infertility, particularly in women over 35 years of age (Menken et al. 1986). In addition, advanced maternal age is associated with a wide range of adverse pregnancy outcomes, including pre‐eclampsia, miscarriage, and chromosomal disorders in fetuses (Frick 2021; Sparić et al. 2024). As the crucial reproductive organ for maintaining female fertility and endocrine function, the ovary is reported to age earlier and faster than most other tissues (Sirard 2022; Wu et al. 2022). Ovarian aging, characterized by the depletion of oocyte number and the decline of oocyte quality, determines the natural loss of fecundity (Balough et al. 2024; Wu, Chen, et al. 2025).

Numerous studies indicate that the age‐related decline in oocyte quality is related to some crucial changes in the oocyte, including failure of spindle assembly, chromosome segregation, and mitochondrial deficiency, ultimately leading to oocyte aneuploidy (Anderson et al. 2025; Charalambous et al. 2023; Zhang et al. 2025). However, these changes are not fully dependent on the oocyte itself. Oocytes are surrounded by layers of tightly packed cumulus granulosa cells (GCs), forming an ensemble called the cumulus‐oocyte complexes (COCs) (Richani et al. 2021; Xie et al. 2023). As adjacent cells to the oocyte, GCs play vital roles in providing metabolic support and facilitating signal communication for the oocyte (Del Bianco et al. 2024; Martinez et al. 2023). As is well known, GCs within this structure regulate oocyte meiosis in response to luteinizing hormone (LH). In addition, the cumulus expansion modulated by prostaglandin E2 (PGE2) or LH is essential for oocyte competence acquisition (Niringiyumukiza et al. 2018; Turathum et al. 2021). It has been reported that enhanced cumulus dilation is closely related to the improvement of oocyte quality (Azari‐Dolatabad et al. 2021; Chaubey et al. 2018). However, limited reports suggest the significant impact of the aged surrounding cellular environment on oocytes during ovarian aging (Bao et al. 2024; Wang et al. 2024). And how aging‐mediated dysregulation of GCs exerts its effects on oocyte quality and the detailed mechanism is still not fully addressed.

We previously demonstrated that downregulation of the mevalonate (MVA) pathway in aged GCs contributes to oocyte meiotic defects (Liu et al. 2023). Preliminary findings further implicated that farnesyl pyrophosphate (FPP)‐mediated protein farnesylation, a downstream MVA pathway, is potentially crucial in the decline of aged oocyte (Liu et al. 2025). Protein farnesylation is a post‐translational lipid modification involving the attachment of a farnesyl group to a C‐terminal cysteine residue, catalyzed by farnesyl transferase (Ashok et al. 2020; Jung and Bachmann 2023). Alterations in genes coding for farnesylated proteins or enzymes involved in farnesylation and maturation underlie severe disease processes, including cancer, neurodegenerative disorders, and premature aging syndromes (Jeong et al. 2022; Juarez and Fruman 2021; Novelli and D'Apice 2012). Notably, a recent study showed that farnesylated compound—farnesol (FOH), which can be catabolized to FPP to increase the farnesylation levels in cells, can prevent aging‐related muscle weakness in mice through enhanced farnesylation of Parkin‐interacting substrate (Bae et al. 2023; Verdaguer et al. 2022). However, whether and how the farnesylation affects oocytes during ovarian aging warrants further investigation.

In the current study, we initially investigated age‐dependent alterations in COCs and observed impaired cumulus expansion and oocyte maturation during ovarian aging, which are accompanied by significantly decreased protein farnesylation in aged GCs. Further study demonstrated that pharmacological inhibition of farnesylation recapitulated the phenotypes of cumulus expansion restriction and oocyte maturation impairment similar to those in aged COCs. Conversely, FOH supplementation—both in aged COCs and aged mice—effectively rescued these deficits through restoring protein farnesylation. Furthermore, a proteomic screen using an alkynyl‐farnesol (alk‐FOH) probe identified prostaglandin E2 synthase 2 (PTGES2) as a key farnesylated protein. We biochemically validated PTGES2 farnesylation and established its essential role in PGE2 production. Mechanistically, downregulated PTGES2 farnesylation in aged GCs reduces PGE2 levels, thereby contributing to impaired cumulus expansion and oocyte maturation during ovarian aging. These findings suggest the critical role of protein farnesylation in GCs on oocyte quality and propose a potential therapeutic strategy for improving the developmental competence of aged oocytes.

## 2 Materials and Methods

### 2.1 Animals

Young (6‐week‐old) and old (8‐month‐old) female C57BL/6 mice were purchased from SPF Biotechnology (China). The 8‐month‐old mice were fed until 10 months as the natural ovarian aging model. All mice had free access to food and water with a 12‐h light–dark cycle and constant temperature. For FOH in vivo supplementation, 9‐month‐old female mice were intraperitoneally injected with 5 mg/kg FOH (Sigma, F203) daily for 30 days. An equivalent volume of normal saline was given in the CTL (Control, CTL) group. All the animal protocols were approved by the Experimental Animal and Welfare Ethics Committee of Nanjing Drum Tower Hospital.

### 2.2 COC Acquisition and In Vitro Maturation (IVM)

The mice were injected with pregnant mare serum gonadotropin (PMSG) (Sansheng Pharmaceuticals, China) (10 IU) for 48 h before being sacrificed. The ovaries were clipped through the abdominal opening, and the peri‐ovarian adipose tissue was removed. The COCs were released from the ovarian antral follicles via a disposable syringe with a 20‐gauge needle under a stereomicroscope. Well‐wrapped COCs were cultured in MEMα maturation medium consisting of 10% fetal bovine serum (Gibco, 10270106), 10 ng/mL epidermal growth factor (Gibco, 53003‐018), and 1.5 IU/mL human chorionic gonadotropin (Sansheng Pharmaceuticals, China) with liquid paraffin oil in an incubator at 37°C with 5% CO_2_ for 14 h. Next, they were transferred into hyaluronidase (Sigma, 9001‐54‐1) to remove the surrounding GCs and calculate the polar body extrusion (PBE) rates of oocytes. The concentration of FOH, FTI‐277 (Aladdin, F331609), and PGE2 (MCE, HY‐101952) used in this study was 50, 50, and 1 μM, respectively. The RI software was used to measure the diameter of COCs individually and record images before and after IVM.

### 2.3 In Vitro Fertilization (IVF) and Embryo Culture

Spermatozoa were collected from the epididymides of 12‐week‐old C57BL/6 male mice and capacitated for 1 h in human tubal fluid medium (HTF, Sigma, MR‐070). Following IVM, the COCs were fertilized by the addition of capacitated sperm for 6 h in a 37°C incubator with 5% CO_2_. Then the fertilized oocytes were transferred to potassium‐supplemented simplex optimized medium (KSOM, Sigma, MR‐106‐D) for subsequent culture. The rate of two‐cell embryo formation was assessed.

### 2.4 Immunofluorescence

Immunofluorescence was performed as we previously described (Guo et al. 2023). Briefly, the samples were fixed with 4% paraformaldehyde (Sigma, 158127) for 30 min. Permeabilization was performed using 0.5% Triton X‐100 (Sigma, T9284) for 20 min, followed by washing three times and blocking with 3% bovine serum albumin (Sigma, 10711454001) for 1 h at room temperature. Mouse anti‐α‐tubulin (Sigma, F2168, 1:700), rabbit polyclonal anti‐calnexin (Proteintech, 10,427‐2‐AP, 1:200) and mouse anti‐PTGES2 (Santa Cruz, sc‐514224, 1:200) were incubated at 4°C overnight. The next day, the samples were washed three times with phosphate‐buffered saline (PBS) (Gibco, C10010500CP) supplemented with 0.05% Tween 20 (PBST) for 5 min. For the co‐staining of PTGES2 and calnexin, donkey anti‐mouse 594 (Invitrogen, A21203, 1:200) and goat anti‐rabbit 488 (Invitrogen, A11008, 1:200) fluorescent secondary antibodies were used for 1 h at room temperature in the dark. After washing again, a DAPI‐containing anti‐fluorescence quencher (Invitrogen, S36968) was used for 10 min at room temperature. Finally, a fluorescence microscope (Leica, DM3000 LED) was used to observe and record the images. For the meiotic defects analysis, chromosome misalignment and spindle abnormalities were assessed as follows: failure of chromosomes to congress at the plate, their disorganization, or any aberration in spindle morphology, including monopolar, multipolar, disorganized, or fragmented microtubule arrays.

### 2.5 Body Weight and Ovarian Index

The mice in each group were weighed and recorded before they were euthanized. After removing the peri‐ovarian adipose tissue, the ovary was weighed and photographed. The ovarian index was determined based on the ovarian wet weight/body weight (mg/g).

### 2.6 KGN Cell Culture, Treatment, and Alk‐FOH Metabolic Labeling

Human KGN cells were cultured in DMEM/F12 (Corning, 10‐092‐CVRC), supplemented with 10% bovine calf serum (BCS) (Sigma, B7446) and 1% penicillin–streptomycin (Gibco, 15140122) in a 37°C incubator with 5% CO_2_. An alk‐FOH chemical reporter was used for metabolic labeling and antagonistic coincubation with natural FOH. When the cells reached approximately 60% confluence, they were divided into the following three groups: 0 μM alk‐FOH, 50 μM alk‐FOH, and 50 μM alk‐FOH + 50 μM FOH (Sigma, F203). After 48 h, the cells were collected separately for further study. For co‐incubation with the inhibitors, KGN cells were treated with 50 μM FOH with or without 25 μM FTI‐277 for 48 h for further detection.

### 2.7 Total Protein and Membrane Protein Isolation

To obtain total protein, the cells were first lysed at 4°C for 30 min using Radio immunoprecipitation assay (NCM Biotech, WB3100) lysis buffer with a protease inhibitor cocktail (Roche, 4693132001). Subsequently, the cell lysate was centrifuged at 4°C and 12,000 rpm for 15 min to obtain the supernatant as the total protein. The membrane protein was extracted with a Membrane and Cytosol Protein Extraction Kit (Beyotime, P0033) according to the manufacturer's instructions. Briefly, membrane protein extraction reagent A, pre‐added with PMSF and a protease inhibitor cocktail, was used to homogenize the cells at 4°C for 30 min. The removal of nuclei and unbroken cells was then carried out by centrifuging at 700 × *g* for 10 min. Next, the supernatant from the previous step was collected for further centrifugation at 12,000 rpm for 30 min to obtain the plasma proteins. Finally, 200 μL of membrane protein extraction reagent B was added to the precipitate for a full vortex for 10 min on ice. The membrane proteins were obtained via centrifugation again at 12,000 rpm for 30 min. The protein concentration was quantified with a BCA assay kit (Thermo Fisher, 23227) following the manufacturer's instructions.

### 2.8 Cu‐Catalyzed Azide‐Alkyne Cycloaddition (CuAAC)/click Reaction and Pull‐Down Assay

The alk‐FOH metabolic labeling and total protein extraction and quantification were performed as described above. Next, a 3 mL protein lysate click mixture was prepared, which consisted of 2520 μL of protein (2.2 mg/mL), 60 μL of azide biotin (0.1 mM, Confluore, 908007‐17‐0), 120 μL of BTTAA‐CuSO4 (2:1, 1 mM: 0.5 mM), and 300 μL of fresh sodium ascorbate (2.5 mM). A shaker was used for the reaction mixture at room temperature for 3 h. Next, the protein sample was precipitated overnight in 24 mL of methanol at −40°C. The following day, the sample was centrifuged and washed twice with pre‐cooled methanol at 5000 rpm for 15 min at 4°C. A total of 1.67 mL of PBS buffer (1.2% SDS in PBS) was used to completely dissolve the precipitates, which needed further air drying at room temperature for 20 min. A total of 100 μL of Pierce high‐capacity streptavidin agarose (Thermo Fisher, 20359) was washed with PBS three times and resuspended in 8.33 mL of PBS. The redissolved proteins were added to the agarose and incubated gently with rotation at room temperature for 4 h. The samples were washed gently with 0.2% SDS in PBS, PBS, and double‐distilled water three times before being centrifuged at 500 rpm for 1 min to remove nonspecific binding proteins. The precipitate was finally transferred into a centrifuge tube with double‐distilled water and mixed with 2× loading buffer (Beyotime, P0015L) in a metallic bath at 95°C for 12 min. The sample was then centrifuged and transferred to a new tube to obtain the protein released from the beads for subsequent western blotting or LC‐MS/MS.

### 2.9 LC–MS/MS Analysis

An Easy‐nLC 1000 (Thermo Fisher) (Buffer A: 0.1% formic acid solution; Buffer B: 0.1% formic acid + 80% acetonitrile solution; Thermo Fisher) was used to detect the enriched protein samples. The protein sample was loaded onto a trap column after equilibration with 95% Buffer A. The separation and analysis were performed via chromatography and MS with a Q Exactive mass 549 spectrometer (Thermo Fisher), respectively. MaxQuant 1.6.14 was used for the raw data search, and the *Homo sapiens* database downloaded from UniProt was used for comparison to obtain the final protein results.

### 2.10 Western Blotting

Western blotting was performed as previously described (Guo et al. 2023). Briefly, after sample quantification, the equivalent protein was first separated via 10% SDS–PAGE and transferred to polyvinylidene difluoride (PVDF) membranes (Millipore, 03010040001). The PVDF membranes were then blocked with 5% nonfat milk for 1 h at room temperature. The primary antibodies used were as follows: rabbit polyclonal anti‐Farnesyl (1:800), rabbit polyclonal anti‐PTGES2 (Proteintech, 10881‐1‐AP, 1:1000), rabbit polyclonal anti‐β‐actin (Abmart, P30002M, 1:2000), rabbit polyclonal anti‐calnexin (Proteintech, 10,427‐2‐AP, 1:1000), and 6 × His, His‐tag mouse monoclonal antibody (Proteintech, 66,005‐1‐Ig, 1:1000) at 4°C overnight. The next day, TBST was used to wash the PVDF membrane three times before incubating with HRP‐conjugated goat anti‐rabbit IgG (Zsbio, ZB‐2301, 1:10000) or HRP‐conjugated goat anti‐mouse IgG (Zsbio, ZB‐2305, 1:10000) at room temperature for 1 h. After washing again, the membrane was developed using enhanced chemiluminescence reagents (Millipore, WBKLS0500). ImageJ was used to estimate the mean gray value.

### 2.11 Plasmid Synthesis and Transfection

The pcDNA 3.0 plasmid (Vector), PTGES2‐overexpressing plasmid (OE), and single‐point mutation plasmid of the CaaX motif in PTGES2 (cysteine at amino acid 16 with serine, C16S) were designed and synthesized by General Biol (China). 293 T cells were cultured with DMEM (Gibco, C11995500BT) with 10% fetal bovine serum and 1% penicillin–streptomycin. When the cells reached 70% confluence, plasmid transfection was performed with Lipofectamine 3000 (Invitrogen, L3000001) following the manufacturer's instructions. In brief, 5 μg of Vector plasmid, OE plasmid, or mutant C16S plasmid was diluted with 500 μL of Opti‐MEM (Gibco, 11,058,021). Lipofectamine 3000 was also diluted with Opti‐MEM consistently. Then, the Lipofectamine 3000 and the plasmid were gently mixed for 15 min at room temperature before being added to the cells. For pull‐down analysis, 50 μM alk‐FOH was also added at the same time as in the OE and C16S groups. For membrane protein detection, 50 μM FOH was added. All the above treatments lasted for 48 h before cell arrest.

### 2.12 Primary Mouse GC Collection and Culture

The mice were sacrificed to obtain ovaries as described above. The ovary was then set into DMEM/F12 medium, and a 1 mL syringe was used to pierce the follicle, releasing the surrounding GCs. A pipette was used to repeatedly blow the released contents. These cells were screened with a 40 μm cell strainer (Corning, 352340). The subsequent centrifugation speed was 1000 rpm for 5 min to discard the supernatant. To obtain enough cells for western blotting, the primary mouse GCs were resuspended in DMEM/F12 supplemented with 10% BCS and 1% penicillin–streptomycin in a 37°C incubator with 5% CO_2_ for culture until 90% confluence for capture.

### 2.13 Enzyme‐Linked Immunosorbent Assay (ELISA)

The PGE2 levels were measured via a PGE2 ELISA Kit (Elabscience, E‐EL‐0034) according to the manufacturer's instructions. In brief, the standards and samples were added to the enzyme‐labeled well plate, and the biotin‐labeled antibody was added to each well immediately for incubation at 37°C for 45 min. Next, the mixture was discarded, and the plate was washed three times. The HRP‐conjugated working solution was added to each well and incubated at 37°C for 30 min before being washed five times as above. Then, the substrate solution was added for 15 min at 37°C in the dark before the stop solution was added. Finally, the OD value was measured with an enzyme‐labeled instrument at 450 nm.

### 2.14 Statistical Analysis

All experiments were performed with at least three independent repeats. The data are shown as means ± SD. Statistical analysis was performed via an unpaired Student's *t*‐test or one‐way ANOVA, and *p* < 0.05 was considered statistically significant. All results were graphed via GraphPad Prism 9.0 (San Diego).

## 3 Results

### 3.1 Cumulus Expansion and Oocyte Maturation Are Impaired During Ovarian Aging

**FIGURE 1:**
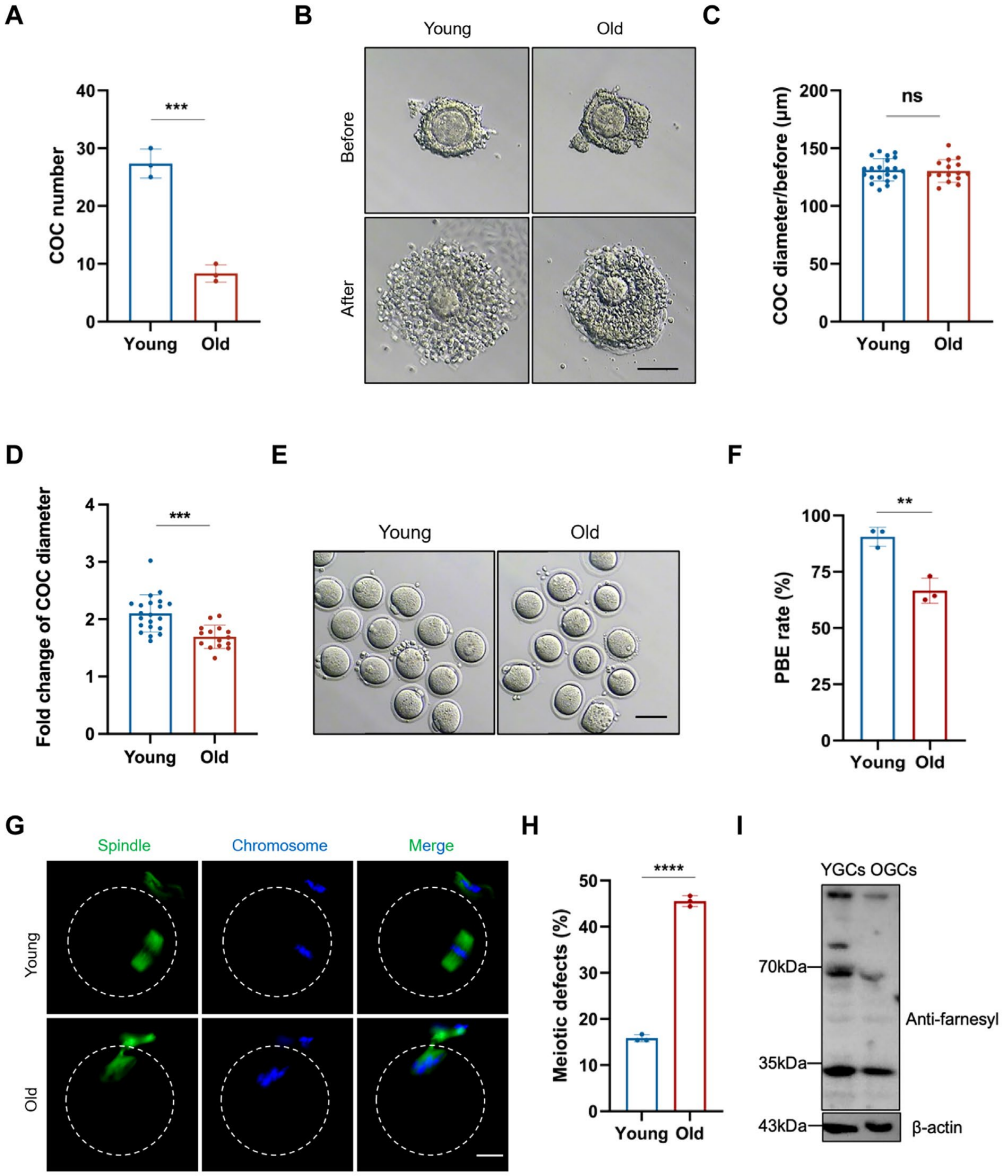
Cumulus expansion and oocyte maturation are impaired during ovarian aging. (A) COCs number from a single mouse in the young and old groups. The young and old mice were based on ages of 6 weeks and 10 months, respectively. (B) Representative COC images before and after cumulus expansion in the young and old groups. Scale bars, 100 μm. (C) COC diameter analysis before cumulus expansion in the young (*n* = 21) and old (*n* = 15) groups. (D) Fold change of COC diameter before and after cumulus expansion in the young (*n* = 21) and old (*n* = 15) groups. (E) Representative oocyte images in the young and old groups. Scale bars, 100 μm. (F) PBE rate of oocytes in the young (*n* = 59) and old (*n* = 43) groups. (G) Representative images of spindle morphologies and chromosome alignment of oocytes in the young and old groups. Scale bars, 25 μm. (H) Meiotic defect rates of oocytes in the young (*n* = 38) and old (*n* = 30) groups. (I) Western blotting showing the farnesylation levels in young and old primary mouse GCs. GCs, granulosa cells. COC, cumulus‐oocyte complex. PBE, polar body extrusion. YGCs, young GCs, OGCs, old GCs. Data are shown as means ± SD from at least three independent repeats. Statistical analysis was performed via an unpaired Student's t‐test. **p < 0.01, ***p < 0.001, ****p < 0.0001, ns, not significant.

To investigate the age‐related changes during ovarian aging, full‐wrapped COCs from young (6‐week‐old) and old (10‐month‐old) mice were collected for IVM. As expected, the number of COCs in the old group was significantly lower than in the young group (8.33 ± 1.53 vs. 27.33 ± 2.52, *p* < 0.001; Figure 1A). Cumulus expansion is essential for oocyte maturation. To assess age‐dependent changes in cumulus expansion, we compared the diameter of young and old COCs before and after IVM. The COC diameters showed no significant difference between the young and old groups before IVM (131.30 ± 9.73 μm vs. 130.50 ± 9.84 μm, *p* > 0.05; Figure 1B,C). After IVM, although both groups exhibited increased COC diameter, the fold‐change in diameter was significantly lower in the old group compared to the young group (1.70 ± 0.20 vs. 2.10 ± 0.32, *p* = 0.0001; Figure 1B,D). This indicates that ovarian aging limits cumulus expansion. We then calculated the PBE rate of oocytes and found a marked decrease in the old group compared to the young group (66.60% ± 5.62% vs. 90.53% ± 4.19%, *p* < 0.01; Figure 1E,F). Notably, most oocytes from young mice displayed barrel‐like spindles with well‐aligned chromosomes, while those from aged mice frequently showed aberrant spindle morphology with misaligned chromosomes (Figure 1G). Quantification analysis revealed a significantly higher rate of meiotic defects in the old group (45.53% ± 1.15% vs. 15.83% ± 0.75%, *p* < 0.0001; Figure 1H).

We previously reported that downregulation of the MVA pathways in aged GCs contributed to oocyte meiotic defects during ovarian aging, and that FPP‐mediated protein farnesylation—a downstream branch of this pathway—may participate in this abnormality. Western blotting of farnesylation levels in young and aged mouse primary GCs further confirmed a significant decline of protein farnesylation in aged GCs (Figure 1I). Collectively, these results suggest decreased farnesylation in aged GCs may be associated with cumulus expansion and oocyte maturation impairment during ovarian aging.

### 3.2 Farnesylation Inhibition Impairs Cumulus Expansion and Oocyte Maturation in Young COCs

**FIGURE 2:**
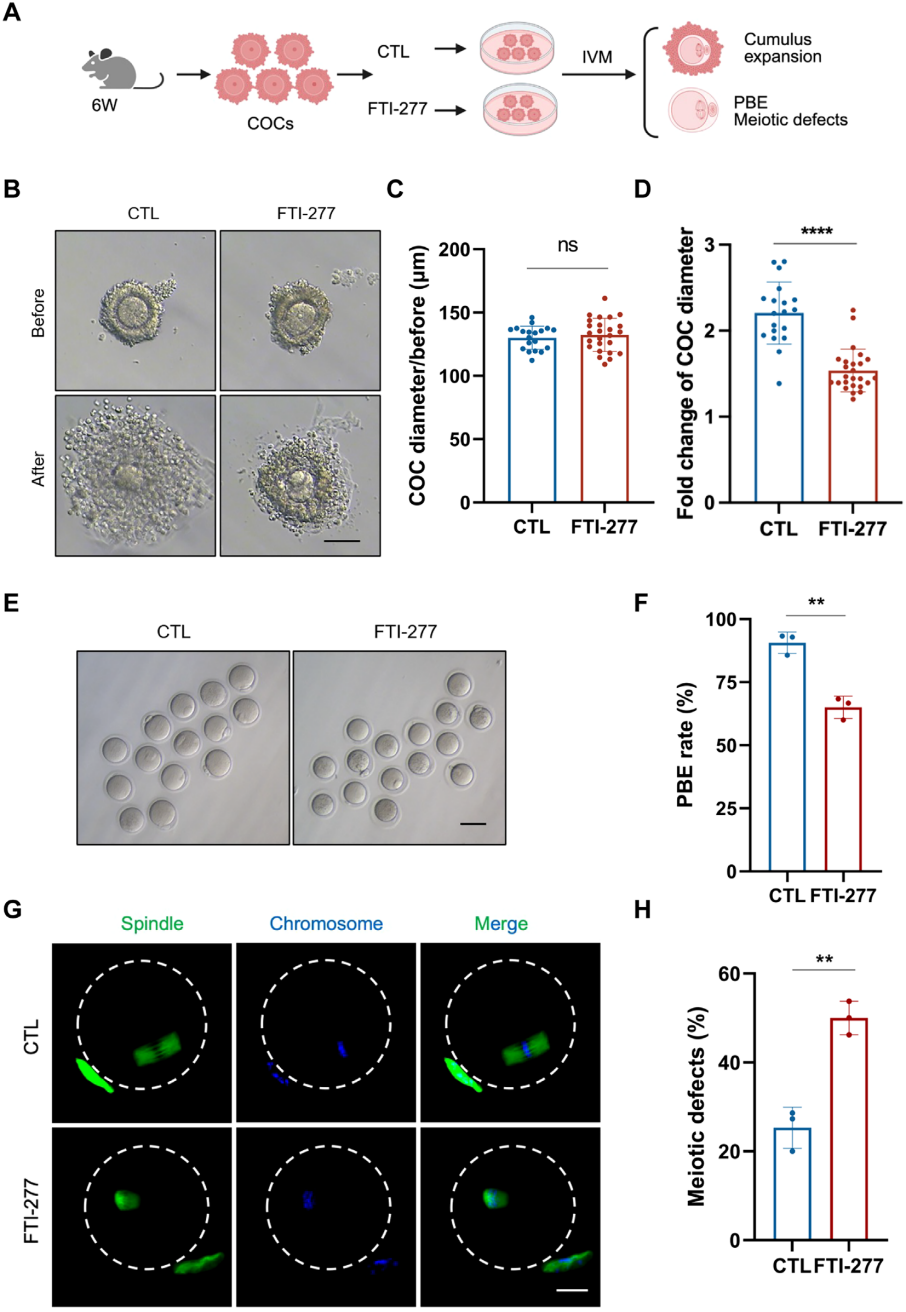
Inhibition of farnesylation leads to cumulus expansion and oocyte maturation impairment in young COCs in vitro. (A) A schematic diagram showing young COCs collection and FTI‐277 treatment for analyzing cumulus expansion and oocyte maturation. (B) Representative COC images before and after cumulus expansion in the CTL and FTI‐277 groups. Scale bars, 100 μm. (C) COC diameter analysis before cumulus expansion in the CTL (*n* = 19) and FTI‐277 (*n* = 25) groups. (D) Fold change of COC diameter before and after cumulus expansion in the CTL (*n* = 19) and FTI‐277 (*n* = 25) groups. (E) Representative oocyte images in the CTL and FTI‐277 groups. Scale bars, 100 μm. (F) PBE rates of oocytes in the CTL (*n* = 36) and FTI‐277 (*n* = 39) groups. (G) Representative images of spindle morphologies and chromosome alignment of oocytes in the CTL and FTI‐277 groups. Scale bars, 25 μm. (H) Meiotic defect rates of oocytes in the CTL (*n* = 33) and FTI‐277 (*n* = 34) groups. COCs, cumulus‐oocyte complexes. IVM, in vitro maturation. PBE, polar body extrusion. CTL (control) group: Young COCs cultured in MEMα. FTI‐277 group: Young COCs cultured in MEM supplemented with 50 μM FTI‐277. Data are shown as means ± SD from at least three independent repeats. Statistical analysis was performed via an unpaired Student's t‐test. **p < 0.01, ****p < 0.0001, ns, not significant.

To further evaluate whether decreased farnesylation would damage cumulus expansion and oocyte maturation, we collected young COCs from 6‐week‐old female mice and cultured them in IVM medium supplemented with FTI‐277 to inhibit farnesylation (Figure 2A). No differences in the COC diameter were observed between groups before IVM (130.10 ± 9.21 μm vs. 132.40 ± 13.06 μm, *p* > 0.05; Figure 2B,C). We found that FTI‐277 treatment markedly suppressed cumulus expansion, resulting in a lower fold change in COC diameter compared to the CTL group (1.54 ± 0.25 vs. 2.21 ± 0.36, *p* < 0.0001; Figure 2B,D). Additionally, we observed a notable decrease in the PBE rate of oocytes in the FTI‐277 group compared with the CTL group (65.03% ± 4.44% vs. 90.63% ± 4.28%, *p* < 0.01; Figure 2E,F). Moreover, FTI‐277 treatment induced spindle abnormalities and chromosome misalignment in oocytes (Figure 2G). Consistently, a higher percentage of meiotic defects was detected in the FTI‐277‐treated group compared to the CTL group (50.00% ± 3.80% vs. 25.30% ± 4.64%, *p* < 0.01; Figure 2H). These findings establish that farnesylation inhibition in young COCs recapitulates the aging‐related phenotypes by impairing cumulus expansion and inducing meiotic defects similar to those observed in aged COCs.

### 3.3 Enhancement of Farnesylation Ameliorates Aged Cumulus Expansion and Oocyte Maturation In Vitro and Vivo

**FIGURE 3:**
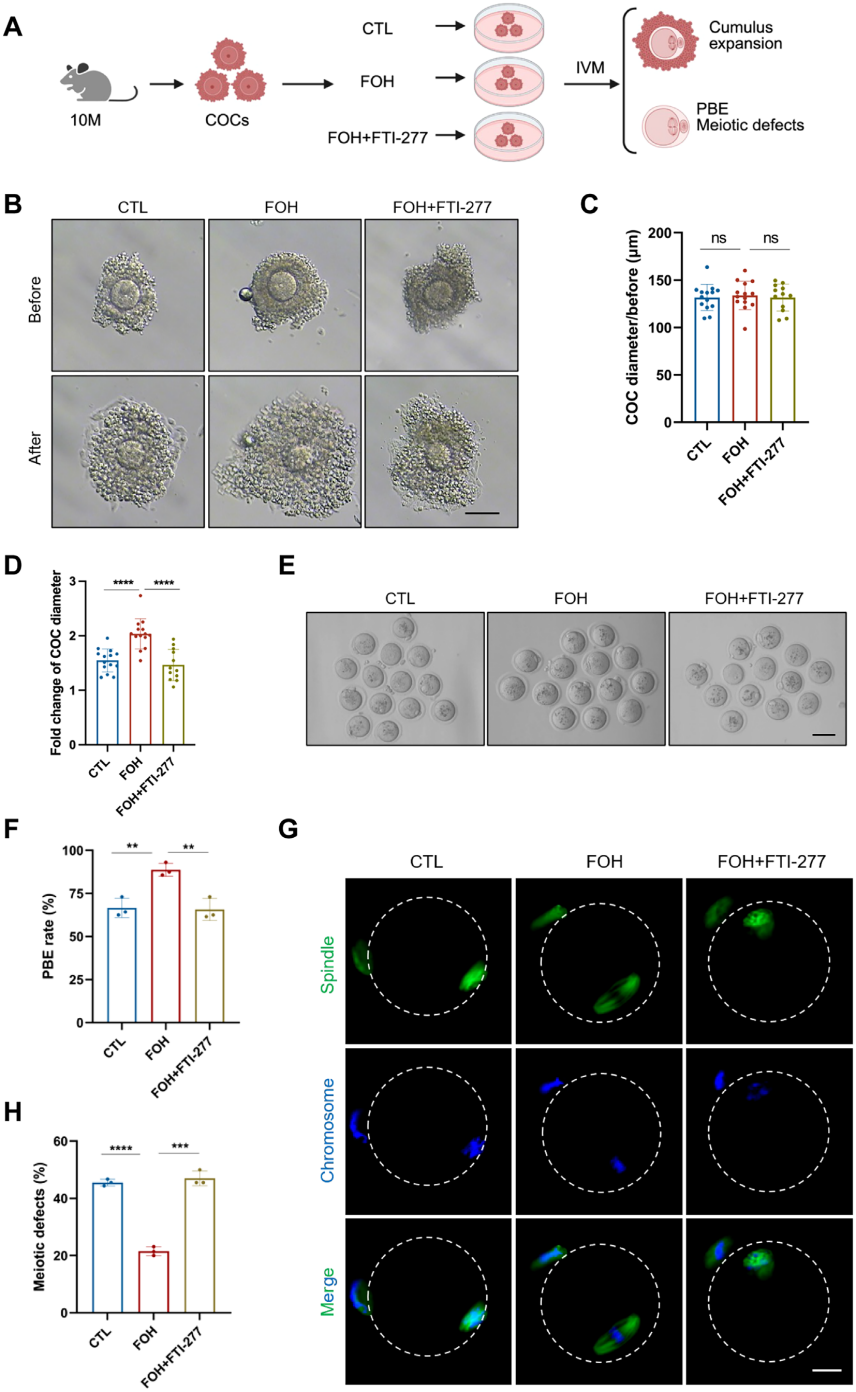
FOH supplementation improves cumulus expansion and oocyte maturation in aged COCs via farnesylation in vitro. (A) A schematic diagram showing old COCs collection and treatment with FOH or FOH + FTI‐277 for analyzing cumulus expansion and oocyte maturation. (B) Representative COC images before and after cumulus expansion in the CTL, FOH, and FOH + FTI‐277 groups. Scale bars, 100 μm. (C) COC diameter analysis before cumulus expansion in the CTL (*n* = 14), FOH (*n* = 14), and FOH + FTI‐277 (*n* = 12) groups. (D) Fold change of COC diameter before and after cumulus expansion in the CTL (*n* = 14), FOH (*n* = 14), and FOH + FTI‐277 (*n* = 12) groups. (E) Representative oocyte images in the CTL, FOH, and FOH + FTI‐277 groups. Scale bars, 100 μm. (F) PBE rates of oocytes in the CTL (*n* = 36), FOH (*n* = 42), and FOH + FTI‐277 (*n* = 40) groups. (G) Representative images of spindle morphologies and chromosome alignment of oocytes in the CTL, FOH, and FOH + FTI‐277 groups. Scale bars, 25 μm. (H) Meiotic defect rates of oocytes in the CTL (*n* = 30), FOH (*n* = 37), and FOH + FTI‐277 (*n* = 44) groups. COCs, cumulus‐oocyte complexes. FOH, farnesol. IVM, in vitro maturation. PBE, polar body extrusion. CTL (control) group: Old COCs cultured in MEMα; FOH group: Old COCs cultured in MEM supplemented with 50 μM FOH; FOH + FTI‐277 group: Old COCs cultured in MEMα supplemented with 50 μM FOH + 25 μM FTI‐277. Data are shown as means ± SD from at least three independent repeats. Statistical analysis was performed via one‐way ANOVA. **p < 0.01, ***p < 0.001, ****p < 0.0001, ns, not significant.

To further investigate the effect of farnesylation on aged COCs, we isolated COCs from 10‐month‐old female mice and cultured them in IVM medium supplemented with FOH. Besides, FTI‐277 was also added to FOH‐supplemented IVM medium to inhibit farnesylation (Figure 3A). We first examined COC diameter, and no difference was detected among the CTL, FOH, and FOH + FTI‐277 groups prior to IVM (131.80 ± 13.84 μm vs. 133.90 ± 15.05 μm, *p* > 0.05; 133.90 ± 15.05 μm vs. 131.70 ± 14.25 μm, *p* > 0.05; Figure 3B,C). Strikingly, FOH treatment substantially restored cumulus expansion with a remarkably higher fold‐change of COC diameter compared to the CTL group (2.04 ± 0.28 vs. 1.55 ± 0.21, *p* < 0.0001; Figure 3B,D). In contrast, FTI‐277 co‐treatment inhibited this FOH‐induced beneficial effect (1.47 ± 0.29 vs. 2.04 ± 0.28, *p* < 0.0001; Figure 3B,D). We further evaluated the effect of farnesylation on oocytes. Our result showed that FOH supplementation significantly increased the PBE rate of aged oocytes via farnesylation compared with the CTL group (88.73% ± 3.80% vs. 66.60% ± 5.62%, *p* < 0.01; Figure 3E,F), however, this improvement was abolished by co‐treatment with FTI‐277 (65.67% ± 6.37% vs. 88.73% ± 3.80%, *p* < 0.01; Figure 3E,F). Spindle structure and chromosome analysis also confirmed that FOH reduced meiotic defects in aged oocytes (21.50% ± 1.55% vs. 45.53% ± 1.15%, *p* < 0.0001; Figure 3G,H), an effect similarly disrupted by FTI‐277 (47.00% ± 2.60% vs. 21.50% ± 1.55%, *p* = 0.0001; Figure 3G,H). These findings demonstrate that farnesylation from GCs mediates the beneficial effect on oocyte maturation, and that enhancing farnesylation improves both cumulus expansion and oocyte maturation in aged COCs in vitro.

To further evaluate the in vivo impact of farnesylation on oocytes, we conducted continuous intraperitoneal injection of FOH to 9‐month‐old mice for 30 days, and an equivalent volume of normal saline was injected as the CTL group (Figure S1A). The results showed a substantial increase in the ovary index in the FOH group compared with the CTL group (0.15 ± 0.03 vs. 0.12 ± 0.02, *p* < 0.01; Figure S1B,C). Moreover, FOH injection rescued cumulus expansion abnormalities in aged COCs, yielding a higher fold change of COC diameter post‐IVM than the CTL group (1.94 ± 0.11 vs. 1.66 ± 0.30, *p* < 0.01; Figure S1D,F), despite comparable diameters before IVM (135.40 ± 10.05 μm vs. 137.30 ± 13.11 μm, *p* > 0.05; Figure S1D,E). We also found that, compared with the CTL group, the PBE rate of oocytes in the FOH group was markedly higher (83.17% ± 3.88% vs. 66.13% ± 3.39%, *p* < 0.01; Figure S1G,H). In addition, FOH injection significantly decreased the meiotic defects of oocytes from aged COCs (24.50% ± 4.27% vs. 43.90% ± 7.88%, *p* < 0.05; Figure S1I,J). These findings confirm that augmentation of farnesylation ameliorates age‐related impairments in cumulus expansion and oocyte maturation in vitro and vivo.

### 3.4 Farnesylated PTGES2 Is Essential for Cumulus Expansion and Oocyte Maturation

**FIGURE 4:**
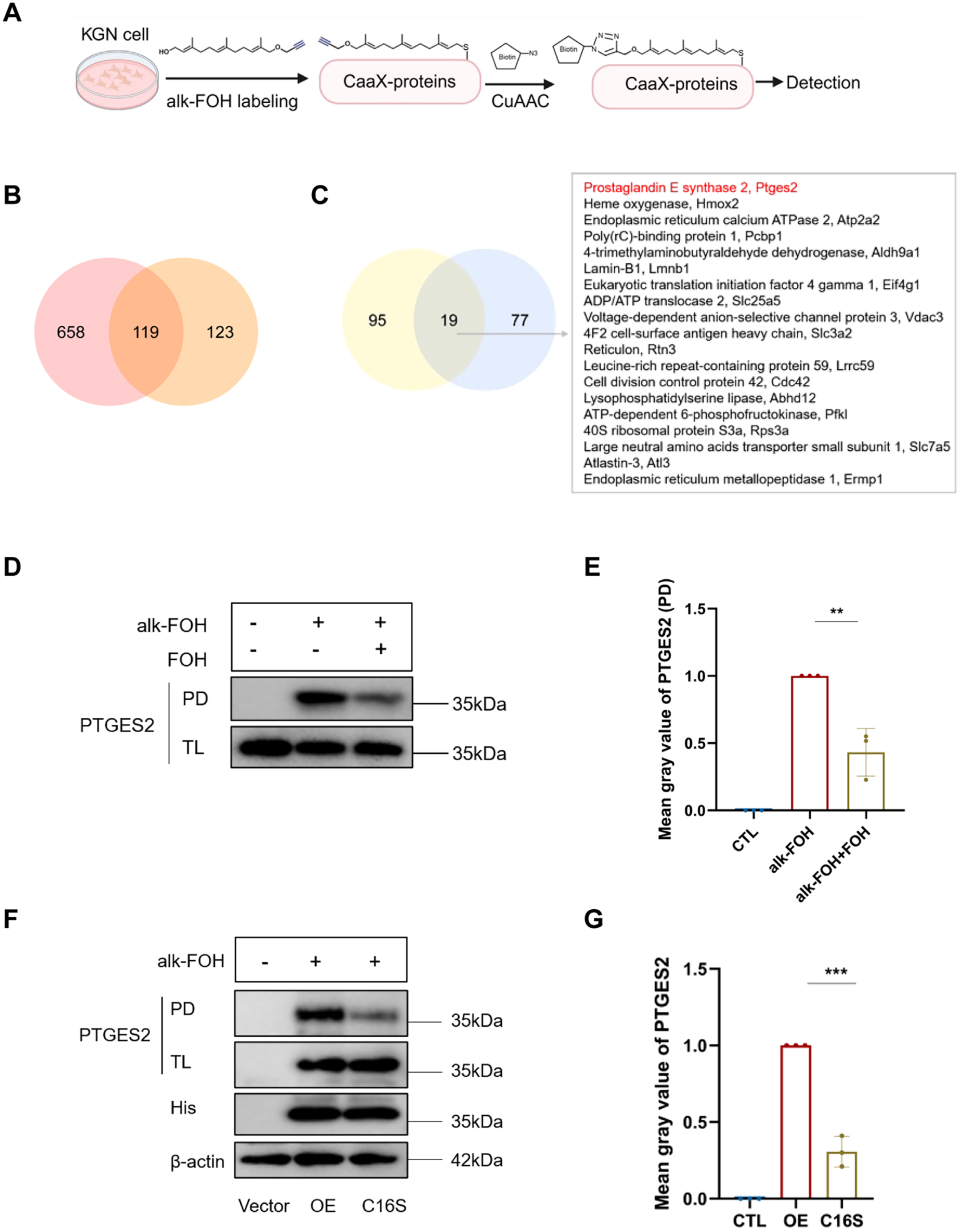
Farnesylated PTGES2 is essential for cumulus expansion and oocyte maturation. (A) Metabolic labeling of cells with alk‐FOH reporter and subsequent CuAAC ligation with bioorthogonal detection tags for proteomics. (B) Overlap of twice‐acquired farnesylated proteomics. (C) Overlap of farnesylated proteins based on our data and the previous report (Charron et al. 2013). (D) Western blotting showing the expression of PTGES2 in the CTL, alk‐FOH, and alk‐FOH + FOH groups. (E) Mean gray value of PTGES2 in the CTL, alk‐FOH, and alk‐FOH + FOH groups. CTL (control) group: KGN cells treated without alk‐FOH or FOH; alk‐FOH group: KGN cells treated with 50 μM alk‐FOH; alk‐FOH + FOH group: KGN cells treated with 50 μM alk‐FOH + 50 μM FOH. (F) Western blotting shows the expression of PTGES2 in the CTL, OE, and C16S groups. (G) Mean gray value of PTGES2 in the CTL, OE, and C16S groups. CTL (control) group: 293 T cells transfected with vehicle plasmid; OE group: 293 T cells transfected with the PTGES2 overexpressing plasmid and treated with 50 μM alk‐FOH; C16S group: 293 T cells transfected with the PTGES2 C16S mutant plasmid and treated with 50 μM alk‐FOH. alk‐FOH, alkynyl‐farnesol. CuAAC, Cu‐catalyzed azide‐alkyne cycloaddition. PTGES2, prostaglandin E2 synthase 2. PD, pull down. TL, Total. His, 6 × hexahistidine tag. Data are shown as means ± SD from at least three independent repeats. Statistical analysis was performed one‐way ANOVA. **p < 0.01, ***p < 0.001.

To comprehensively identify farnesylation candidates in GCs, we employed the alk‐FOH chemical reporter, a sensitive tool for biochemical analysis of farnesylated proteins. In brief, the human granulosa‐like tumor cell line KGN cells were incubated with or without alk‐FOH, and then total cellular protein was subjected to click chemistry ligation with azide‐biotin (CuAAC), followed by LC‐MS/MS analysis (Figure 4A). A total of 119 candidate proteins were identified based on twice‐acquired proteomic data (Figure 4B). After filtering out 23 low‐confidence hits, a total of 96 proteins associated with the alk‐FOH group were identified. By intersecting this dataset with a previously published 114 farnesylated proteins (Charron et al. 2013), we identified 19 high‐confidence candidate proteins (Figure 4C). Among these, PTGES2 emerged as a prime candidate due to its established enzymatic role in PGE2 synthesis, which is a key mediator of cumulus expansion and oocyte maturation.

To validate PTGES2 as a farnesylated protein, we performed affinity enrichment of the alk‐FOH‐labeled proteins. The result showed that PTGES2 can be labeled with alk‐FOH, and the labeling was sensitive to competition with natural FOH (Figure 4D,E). As farnesylation requires a C‐terminal CaaX motif (where C denotes the critical cysteine residue), we generated a C16S point mutant (cysteine 16 mutated to serine) to ablate the putative farnesylation site. Western blotting analysis further confirmed that wild‐type PTGES2 undergoes farnesylation, while the C16S mutation abolished this modification (Figure 4F,G). Collectively, these results identify PTGES2 as a farnesylated protein in GCs, suggesting its essential role in cumulus expansion and oocyte maturation.

### 3.5 Farnesylation Enables PTGES2 Localization to Endoplasmic Reticulum (ER) and Facilitates PGE2 Production

**FIGURE 5:**
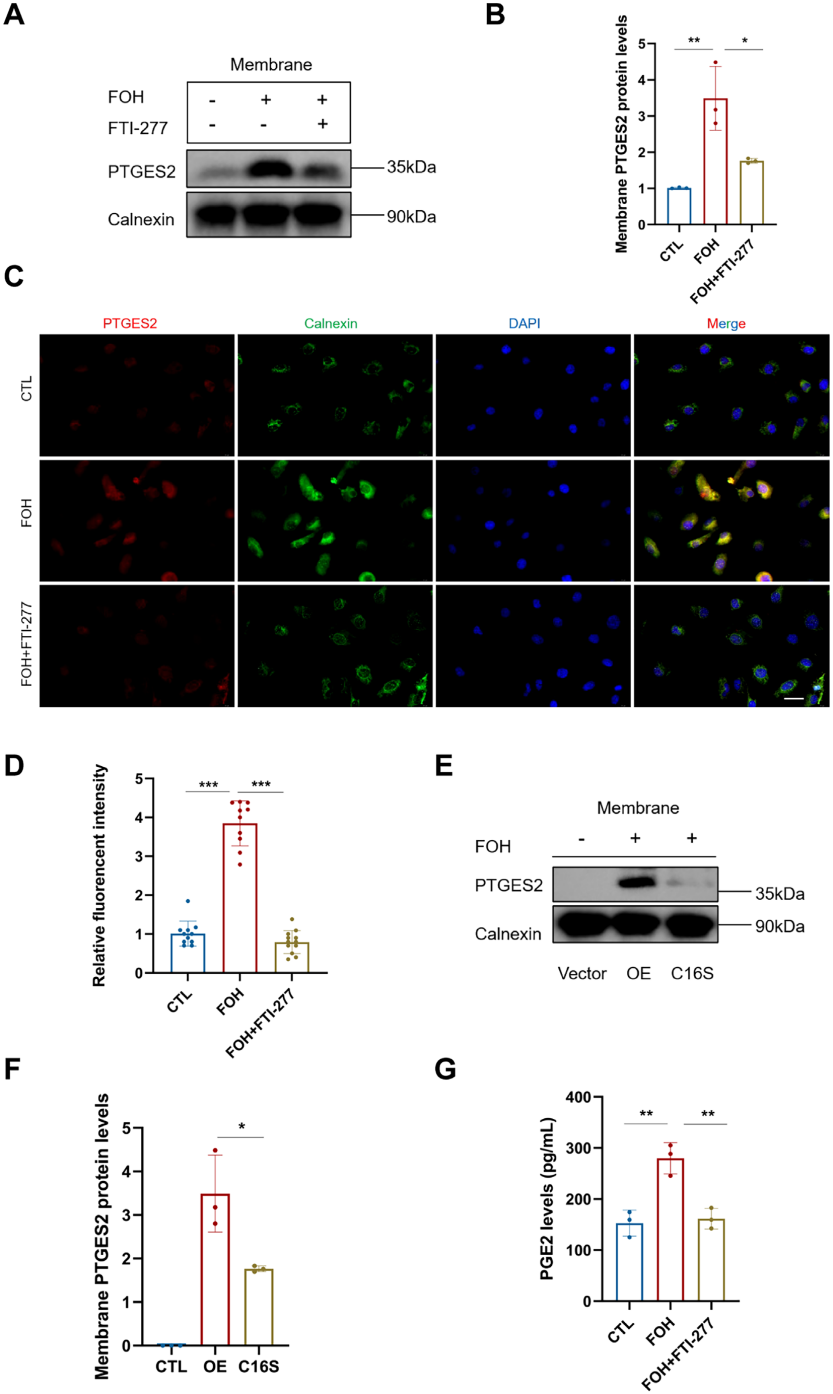
PTGES2 farnesylation facilitates localization to the endoplasmic reticulum and PGE2 production. (A) Western blotting showing PTGES2 expression in the membrane fractions in the CTL, FOH, and FOH + FTI‐277 groups. (B) Mean gray value of PTGES2 in the CTL, FOH, and FOH + FTI‐277 groups. (C) Immunofluorescence co‐staining of PTGES2 and calnexin in the CTL, FOH, and FOH + FTI‐277 groups. Scale bars, 10 μm. (D) Relative fluorescent intensity of PTGES2 and calnexin co‐staining in the CTL, FOH, and FOH + FTI‐277 groups. CTL (control) group: KGN cells without treatment; alk‐FOH group: KGN cells treated with 50 μM alk‐FOH; alk‐FOH + FOH group: KGN cells treated with 50 μM alk‐FOH + 50 μM FOH. (E) Western blotting showing PTGES2 expression in the membrane fractions in the CTL, OE, and C16S groups. (F) Mean gray value of PTGES2 in the CTL, OE, and C16S groups. CTL (control) group: 293 T cells transfected with vehicle plasmid; OE group: 293 T cells transfected with the PTGES2 overexpressing plasmid and treated with 50 μM alk‐FOH; C16S group: 293 T cells transfected with the PTGES2 C16S mutant plasmid and treated with 50 μM alk‐FOH. (G) ELISA showing PGE2 levels in the conditional culture media of KGN cells in the CTL, FOH, and FOH + FTI‐277 groups. FOH, farnesol. PTGES2, prostaglandin E2 synthase 2. OE, PTGES2‐overexpression plasmid. C16S, single‐point mutation plasmid of the CaaX motif in PTGES2. Data are shown as means ± SD from at least three independent repeats. Statistical analysis was performed via one‐way ANOVA. *p < 0.05, **p < 0.01, ***p < 0.001.

As a type of protein lipid modification, farnesylation enhances the hydrophobicity of proteins and promotes their membrane localization. We therefore assessed whether farnesylation promotes PTGES2 membrane localization. Western blotting analysis showed that FOH treatment considerably increased PTGES2 membrane localization via farnesylation (Figure 5A,B). Conversely, farnesyltransferase inhibition with FTI‐277 abrogated FOH‐induced membrane anchoring of PTGES2 (Figure 5A,B). Considering PTGES2 depends on ER localization for PGE2 synthesis, we assessed its colocalization with the ER marker calnexin. Immunofluorescence staining revealed that FOH enhanced the localization of PTGES2 to the ER via farnesylation, while FTI‐277 significantly reduced this localization (Figure 5C,D).

To further investigate the role of the farnesylation motif, we mutated the critical cysteine residue. Compared to the wild‐type PTGES2 overexpressing group, the mutant C16S significantly decreased PTGES2 expression on the membrane, demonstrating that PTGES2 has an effective farnesylated functional motif to promote its ER localization (Figure 5E,F). We also detected PGE2 levels in the conditional culture media after FOH or FTI‐277 supplementation. The result showed that FOH significantly increased PGE2 synthesis, while FTI‐277 supplementation reduced PGE2 production (Figure 5G). Together, these findings demonstrate that farnesylation of PTGES2 regulates its ER localization and is required for PGE2 production.

### 3.6 PTGES2 Farnesylation Improves Cumulus Expansion and Oocyte Maturation Through PGE2

**FIGURE 6:**
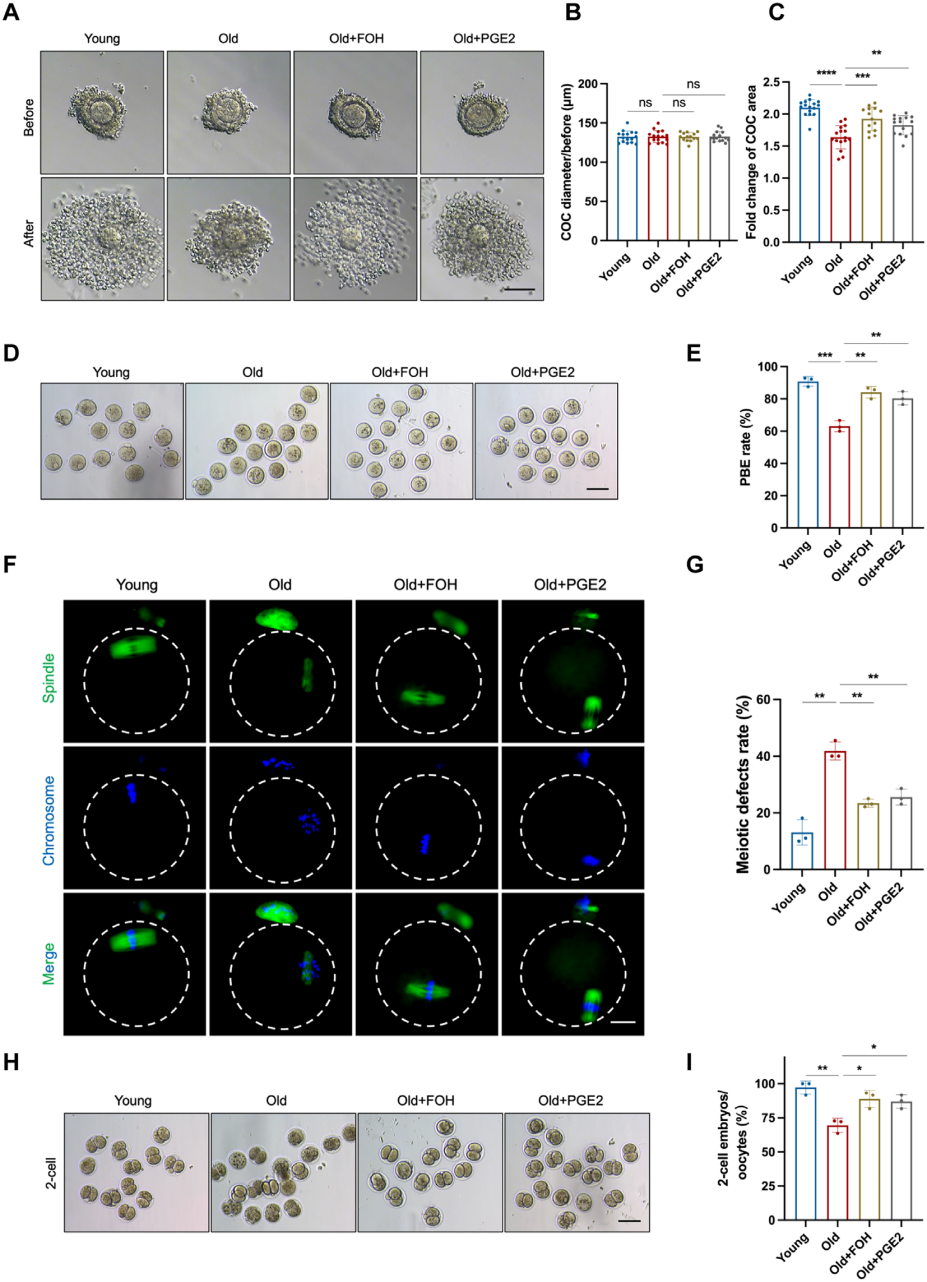
Farnesylation of PTGES2 improves aged cumulus expansion and oocyte maturation through PGE2. (A) Representative COC images before and after cumulus expansion in the Young, Old, Old + FOH, and Old + PGE2 groups. Scale bars, 100 μm. (B) COC diameter before cumulus expansion in the Young (*n* = 16), Old (*n* = 16), Old + FOH (*n* = 14), and Old + PGE2 (*n* = 15) groups. (C) Fold change of COC diameter before and after cumulus expansion in the Young (*n* = 16), Old (*n* = 16), Old + FOH (*n* = 14), and Old + PGE2 (*n* = 15) groups. (D) Representative oocyte images in the Young, Old, Old + FOH, and Old + PGE2 groups. Scale bars, 100 μm. (E) PBE rates of oocytes in the Young (*n* = 46), Old (*n* = 41), Old + FOH (*n* = 40), and Old + PGE2 (*n* = 45) groups. (F) Representative images of spindle morphologies and chromosome alignment of oocytes in the Young, Old, Old + FOH, and Old + PGE2 groups. Scale bars, 25 μm. (G) Meiotic defect rates of oocytes in the Young (*n* = 34), Old (*n* = 36), Old + FOH (*n* = 31), and Old + PGE2 (*n* = 33) groups. (H) Representative images of 2‐cell embryos from MIIOs in the Young, Old, Old + FOH, and Old + PGE2 groups. (I) The 2‐cell embryos rate in the Young (*n* = 46), Old (*n* = 40), Old + FOH (*n* = 38), and Old + PGE2 (*n* = 36). COCs, cumulus‐oocyte complexes. FOH, farnesol. PGE2, prostaglandin E2. IVM, in vitro maturation, PBE, polar body extrusion. Young group: Young COCs cultured in MEMα; Old group: Old COCs cultured in MEMα; Old + FOH group: Old COCs cultured in MEM supplemented with 50 μM FOH; Old + PGE2 group: Old COCs cultured in MEM supplemented with 1 μM PGE2. Data are shown as means ± SD from at least three independent repeats. Statistical analysis was performed via one‐way ANOVA. *p < 0.05, **p < 0.01, ***p < 0.001, ***p < 0.0001, ns, not significant.

To further determine whether PTGES2 farnesylation ameliorates age‐related deficits in cumulus expansion and oocyte maturation via PGE2, we treated aged COCs with either PGE2 or FOH. Before IVM, no significant differences in COC diameter were observed among groups (132.30 ± 7.37 μm vs. 132.60 ± 7.65 μm, *p* > 0.05; 132.60 ± 7.65 μm vs. 132.20 ± 5.39 μm, *p* > 0.05; 132.60 ± 7.65 μm vs. 132.80 ± 6.51 μm, *p* > 0.05; Figure 6A,B). After IVM, however, both FOH and PGE2 significantly restored cumulus expansion in aged COCs. This was evidenced by a greater fold change in COC diameter (1.93 ± 0.18 vs. 1.64 ± 0.18, *p* < 0.001; 1.83 ± 0.15 vs. 1.64 ± 0.18, *p* < 0.01; Figure 6A,C). Similarly, PGE2 supplementation markedly enhanced the PBE rate in aged oocytes (80.37% ± 4.06% vs. 63.07% ± 3.39%, *p* < 0.01; Figure 6D–E), mirroring the effect of FOH (84.13% ± 3.61% vs. 63.07% ± 3.39%, *p* < 0.01; Figure 6D–E). Furthermore, PGE2 effectively reduced meiotic defects in aged oocytes, similar to FOH treatment (25.57% ± 2.79% vs. 41.83% ± 3.18%, *p* < 0.01; 23.43% ± 1.43% vs. 41.83% ± 3.18%, *p* < 0.01; Figure 6F–G). We further performed IVF to compare the early embryonic development. The result showed that both PGE2 and FOH significantly improved the two‐cell embryo rate of aged COCs (87.00% ± 4.97% vs. 69.50% ± 5.12%, *p* < 0.01; 88.93% ± 6.23% vs. 69.50% ± 5.12%, *p* < 0.01; Figure 6H,I). Together, these results demonstrate that PGE2 recapitulates the beneficial effects of FOH. It effectively restores the age‐related declines in both cumulus expansion and oocyte maturation. These findings strongly support that PTGES2 farnesylation enhances oocyte quality, putatively through PGE2.

## 4 Discussion

The trend of delayed childbearing presents a significant health challenge for women of advanced reproductive age (Balasch and Gratacós 2011; Beaujouan et al. 2019; Gosden and Rutherford 1995). Age‐related deterioration in oocyte quality constitutes a primary cause of declining fertility. However, the underlying mechanisms remain incompletely understood, and effective interventions are limited. Our study establishes farnesylation as a critical regulatory mechanism underlying impaired cumulus expansion and oocyte maturation during ovarian aging. We identify PTGES2 as a key farnesylation target and demonstrate that age‐related decline in its modification disrupts ER localization, leading to reduced PGE2 biosynthesis. Consequently, this impairment compromises PGE2‐dependent signaling, contributing to defective cumulus expansion and oocyte maturation in the aging ovary.

Cumulus expansion, triggered by the luteinizing hormone surge, facilitates oocyte meiotic resumption and is a critical determinant of oocyte competence and subsequent embryo development (Turathum et al. 2021). It has been reported that enhanced cumulus expansion correlates with improved oocyte quality and blastocyst development (Azari‐Dolatabad et al. 2021; Chaubey et al. 2018; Lee et al. 2020). We currently observed that cumulus expansion and oocyte maturation were impaired in aged COCs. Consistent with our results, a recent study also reported age‐related defects in cumulus expansion (Babayev et al. 2023). These findings highlighted that there are significant age‐dependent differences in cumulus expansion and oocyte maturation during the ovarian aging process. Notably, impaired cumulus expansion likely disrupts the communication between aged GCs and oocytes, contributing to oocyte maturation failure.

The decline in oocyte quality with age is increasingly viewed not merely as an intrinsic oocyte defect, but as a failure of the supportive follicular microenvironment (Wang et al. 2024; Wu, Liu, and Ding 2025). This microenvironment is shaped by a crucial bidirectional communication between the oocyte and its surrounding somatic cells. Classically, it is well established that oocyte‐to‐granulosa cell signaling, mediated by oocyte‐secreted factors, such as GDF9 and BMP15, stimulates granulosa cell functions including proliferation and cholesterol biosynthesis, dominating early folliculogenesis (Eppig 2001; Su et al. 2009; Wang et al. 2025). However, reciprocal support from granulosa cells to the oocyte is equally vital, particularly for late‐stage oocyte maturation and in the context of aging, where this support may falter. This concept of somatic cell support is not merely descriptive but reveals a therapeutically promising plasticity in the aged ovary. For example, a recent study reported that exposure to the young follicular microenvironment can rejuvenate aged oocyte quality, highlighting the supporting role of surrounding follicular somatic cells in oocytes (Wang et al. 2024). Our previous work implicated that reduced farnesylation in aged GCs may contribute to oocyte meiotic dysfunction (Liu et al. 2025, 2023). The present study directly addresses this gap by elucidating a specific mechanism of granulosa cell support. Currently, we further elucidate that decreased farnesylation in aged GCs is associated with impaired cumulus expansion and oocyte maturation. Inhibition of farnesylation recapitulated the phenotypes of cumulus expansion restriction and oocyte maturation impairment in aged COCs. Conversely, we propose an effective strategy to improve age‐related deficits in cumulus expansion and oocyte maturation by enhancing protein farnesylation through FOH treatment both in vitro and in vivo. These highlight farnesylation in GCs as a promising therapeutic target for mitigating ovarian aging. Limited studies have also reported farnesylation in the aging process. For example, a previous study reported that the accumulation of farnesylated prelamin A intermediates led to premature aging (Reddy and Comai 2012). Another recent study found that enhanced farnesylation of the Parkin‐interacting substrate prevented aging‐related muscle weakness in mice (Bae et al. 2023). Our findings further expand the understanding the regulatory role of protein farnesylation in ovarian aging.

The improved alk‐FOH chemical reporter allows large‐scale profiling of farnesylated proteins (Charron et al. 2013, 2011). In agreement with previous farnesylated protein data, we currently identified farnesylated PTGES2, based on the alk‐FOH (Charron et al. 2013). Farnesylation facilitates membrane anchoring, promoting protein‐membrane interactions and intracellular signaling (Charron et al. 2013; Cox and Der 1992). We confirmed that PTGES2 farnesylation contributed to its ER localization, and consequently, to increased PGE2 production. The well‐established role of PGE2 in oocyte meiotic maturation, cumulus expansion, and follicle rupture (Ben‐Ami et al. 2006; Niringiyumukiza et al. 2018) suggests that age‐related reduction in PTGES2 farnesylation in granulosa cells mechanistically underlies compromised PGE2 signaling during ovarian aging. While our mouse model offers important mechanistic insights, future studies using human tissues will be essential to validate the translational potential of these findings. Collectively, our work elucidates how protein farnesylation in GCs regulates oocyte function, revealing novel mechanisms of granulosa‐oocyte communication and proposing a potential therapeutic strategy to counteract age‐related decline in oocyte quality.

## Author Contributions

Haixiang Sun and Lijun Ding designed the project. Sainan Zhang performed the main experiments and data analysis. Jiahui Qi and Chuanming Liu helped with the crucial experiments. Huidan Zhang, Bichun Guo, Die Wu, Yicen Liu, Nannan Kang, Yang Zhang, Xin Zhen, and Guijun Yan assisted in the methodology. Sainan Zhang wrote the original manuscript, and Chuanming Liu and Lijun Ding revised the manuscript. Haixiang Sun, Lijun Ding, and Chaojun Li provided crucial advice and support. All authors read and approved the final manuscript.

## Funding

This work was financially supported by grants from the Youth Science Fund Project (A category) of the National Natural Science Foundation of China (82525027), the National Natural Science Foundation of China (82271671, 82030040), the Key International Cooperation Project of National Natural Science Foundation of China (82320108008), Jiangsu Frontier Technology R&D Program (Health and Wellness Sector) (BF2025626), Nanjing Drum Tower Hospital Academic Innovation Peak Fund (2024‐DF‐02), and the Clinical Trials from Nanjing Drum Tower Hospital (2023‐LCYJ‐MS‐05).

## Conflicts of Interest

The authors declare no conflicts of interest.

## Supporting information


**Figure S1:** FOH supplementation improves cumulus expansion and oocyte maturation in aged mice via farnesylation in vivo. (A) A schematic diagram showing the FOH or NS injection and COCs collection for analyzing cumulus expansion and oocyte maturation. (B) Representative ovary micrographs in the CTL and FOH groups. Scale bars, 1 mm. (C) Ovary index in the CTL (*n* = 12) and FOH (*n* = 12) groups. (D) Representative COC images in the CTL and FOH groups. Scale bars, 100 μm. (E) COC diameter analysis before cumulus expansion in the CTL (*n* = 13) and FOH (*n* = 13) groups. (F) Fold change of COC diameter before and after cumulus expansion in the CTL (*n* = 13) and FOH (*n* = 13) groups. (G) Representative oocyte images in the CTL and FOH groups. Scale bars, 100 μm. (H) PBE rates of oocytes in the CTL (*n* = 32) and FOH (*n* = 42) groups. (I) Representative images of spindle morphologies and chromosome alignment of oocytes in the CTL and FOH groups. Scale bars, 25 μm. (J) Meiotic defect rates of oocytes in the CTL (*n* = 29) and FOH (*n* = 31) groups. NS, normal saline. FOH, farnesol. IVM, in vitro maturation. PBE, polar body extrusion. CTL (control) group: 9‐month‐old female mice were injected with NS; FOH group: 9‐month‐old female mice were injected with 5 mg/kg FOH. Data are shown as means ± SD from at least three independent repeats. Statistical analysis was performed via an unpaired Student's t‐test. *p < 0.05, **p < 0.01, ns, not significant.

## Data Availability

The data that support the findings of this study are available from the corresponding author as needed.
