# Supplementary material for: Decreased PTGES2 Farnesylation in Granulosa Cells Compromises PGE2‐Dependent Cumulus Expansion and Oocyte Maturation During Ovarian Aging
Source: Aging Cell. 2026 Jan 11;25(2):e70374. doi: 10.1111/acel.70374 (PMC12793032; doi:10.1111/acel.70374)
Supplement: Supplementary file 1 — Figure S1: FOH supplementation improves cumulus expansion and oocyte maturation in aged mice via farnesylation in vivo. (A) A schematic diagram showing the FOH or NS injection and COCs collection for analyzing cumulus expansion and oocyte maturation. (B) Representative ovary micrographs in the CTL and FOH groups. Scale bars, 1 mm. (C) Ovary index in the CTL (n = 12) and FOH (n = 12) groups. (D) Representative COC images in the CTL and FOH groups. Scale bars, 100 μm. (E) COC diameter analysis before cumulus expansion in the CTL (n = 13) and FOH (n = 13) groups. (F) Fold change of COC diameter before and after cumulus expansion in the CTL (n = 13) and FOH (n = 13) groups. (G) Representative oocyte images in the CTL and FOH groups. Scale bars, 100 μm. (H) PBE rates of oocytes in the CTL (n = 32) and FOH (n = 42) groups. (I) Representative images of spindle morphologies and chromosome alignment of oocytes in the CTL and FOH groups. Scale bars, 25 μm. (J) Meiotic defect rates of oocytes in the CTL (n = 29) and FOH (n = 31) groups. NS, normal saline. FOH, farnesol. IVM, in vitro maturation. PBE, polar body extrusion. CTL (control) group: 9‐month‐old female mice were injected with NS; FOH group: 9‐month‐old female mice were injected with 5 mg/kg FOH. Data are shown as means ± SD from at least three independent repeats. Statistical analysis was performed via an unpaired Student's t‐test. *p < 0.05, **p < 0.01, ns, not significant. [file ACEL-25-e70374-s001.docx]

**Supporting Information**


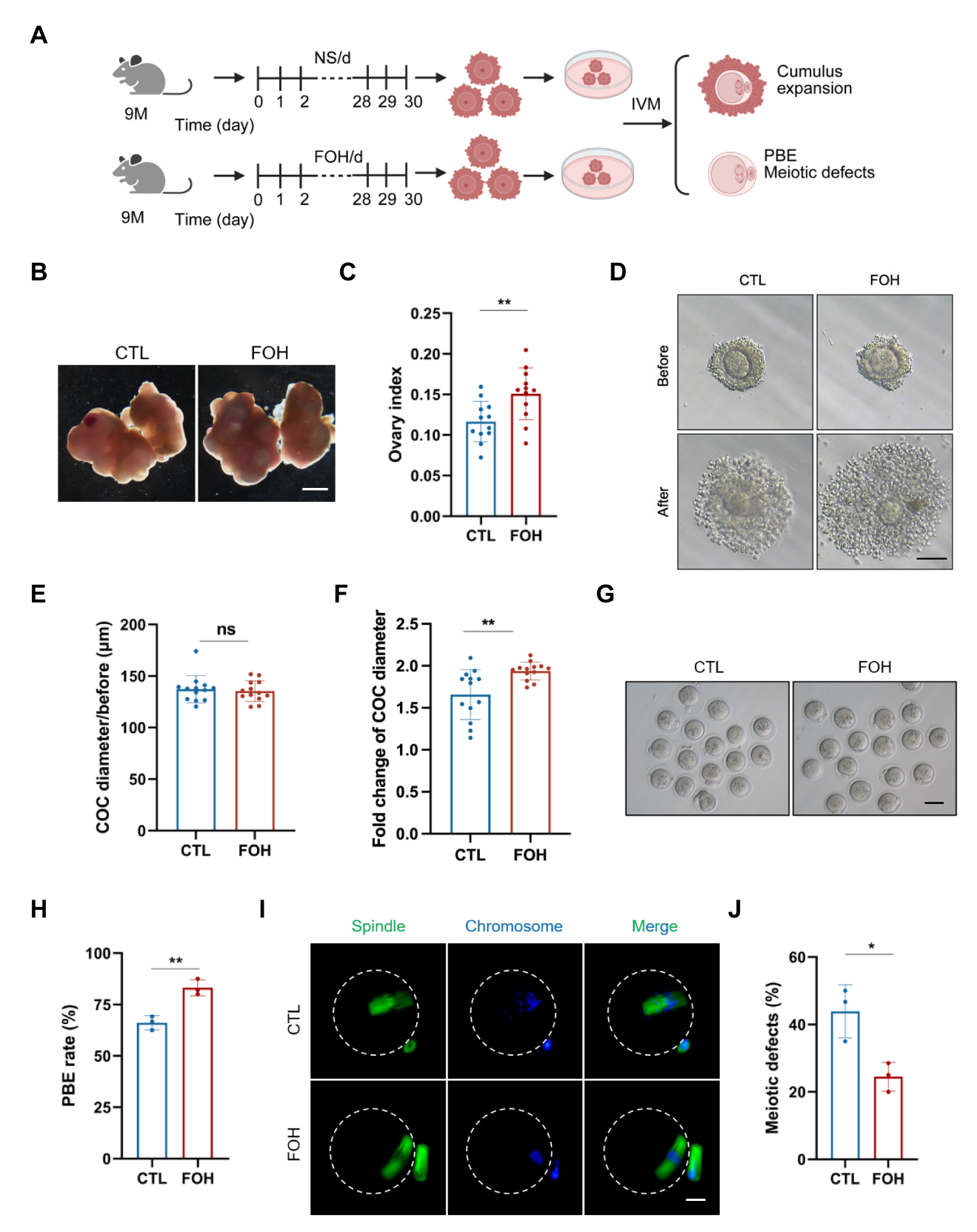


**Supporting information Figure 1** FOH supplementation improves cumulus expansion and oocyte maturation in aged mice via farnesylation *in vivo*. (A) A schematic diagram showing the FOH or NS injection and COCs collection for analyzing cumulus expansion and oocyte maturation. (B) Representative ovary micrographs in the CTL and FOH groups. Scale bars, 1 mm. (C) Ovary index in the CTL (n=12) and FOH (n=12) groups. (D) Representative COC images in the CTL and FOH groups. Scale bars, 100 μm. (E) COC diameter analysis before cumulus expansion in the CTL (n=13) and FOH (n=13) groups. (F) Fold change of COC diameter before and after cumulus expansion in the CTL (n=13) and FOH (n=13) groups. (G) Representative oocyte images in the CTL and FOH groups. Scale bars, 100 μm. (H) PBE rates of oocytes in the CTL (n=32) and FOH (n=42) groups. (I) Representative images of spindle morphologies and chromosome alignment of oocytes in the CTL and FOH groups. Scale bars, 25 μm. (J) Meiotic defect rates of oocytes in the CTL (n=29) and FOH (n=31) groups. NS, normal saline. FOH, farnesol. IVM, *in vitro* maturation. PBE, polar body extrusion. CTL (control) group: 9-month-old female mice were injected with NS; FOH group: 9-month-old female mice were injected with 5 mg/kg FOH. Data are shown as means ± SD from at least three independent repeats. Statistical analysis was performed via an unpaired Student’s t-test. **P* < 0.05, ***P* < 0.01, ns, not significant.
